# Characteristics of the ideal healthcare services to meet adolescents' mental health needs: A qualitative study of adolescents' perspectives

**DOI:** 10.1111/hex.13600

**Published:** 2022-09-08

**Authors:** Laia G. Meldahl, Lou Krijger, Maren M. Andvik, Nicole E. Cardenas, Oliver Cuddeford, Samuel Duerto, Julia R. Game, Maya Ibenfeldt, Murad Mustafa, Mathias Tong, Petter Viksveen

**Affiliations:** ^1^ Department for Quality and Health Technology, SHARE—Centre for Resilience in Healthcare, Faculty of Health Sciences University of Stavanger Stavanger Norway; ^2^ ESCP Europe (Business Management) Ecole Supérieure de Commerce de Paris Paris France; ^3^ School of Medical, Veterinary and Life Sciences (Zoology) University of Glasgow Glasgow Scotland; ^4^ School of Psychology (Psychology) University of Aberdeen Aberdeen Scotland; ^5^ Faculty of Art Design and Architecture (Architecture) University of Huddersfield Huddersfield UK; ^6^ Faculty of Philosophy, Theology and Religious Studies (Philosophy, Politics and Societies) Radboud University Nijmegen The Netherlands; ^7^ Faculty of Medicine Pomeranian Medical University Szczecin Poland; ^8^ Faculty of Biology, Medicine and Health (Pharmacology) University of Manchester Manchester UK; ^9^ Faculty of Health Sciences (Nursing) University of Stavanger Stavanger Norway; ^10^ Department of Chemical Engineering and Analytical Science (Chemical Engineering) University of Manchester Manchester UK

**Keywords:** adolescents, codesign, health services design, involvement, mental health, youth

## Abstract

**Introduction:**

Despite increased focus on development of mental health services worldwide, many adolescents still hesitate to reach out to the services when they need them. This might be linked to the lack of adolescent involvement in the development of services. This study aimed to explore adolescents' perspectives on the ideal healthcare services to meet their mental health needs.

**Methods:**

A two‐stage qualitative study was carried out to explore the perspectives of adolescents on the healthcare services to support their mental health. In the first stage, data from 295 adolescents attending a mental health seminar were analysed using qualitative content analysis. In the second stage, in‐depth perspectives of 10 adolescent coresearchers were gathered through self‐reflection and group discussions to further explore the categories developed in the first stage. Thematic analysis was used to develop the themes answering the research question. Ten adolescent coresearchers planned the study, collected and analysed data and authored the manuscript, with the support of a senior researcher.

**Results:**

Five themes describe adolescents' perspectives on the ideal healthcare services to meet their mental health needs: (1) Culturally Sensitive and Responsive; (2) Communication of Information; (3) Easy Access; (4) Variety of Support; and (5) Consistency. Culturally Sensitive and Responsive services influenced all other themes. The themes describe suggestions for mental health service improvement, including how to manage the barriers that adolescents face to receive help from the mental health services.

**Discussion:**

This study highlights the importance of culturally sensitive and responsive services. It raises the need for an expanded definition of culture going beyond nationality and ethnic background. Adolescents need flexible services that meet their individual mental health needs. This has implications for practitioners, educators, system organizers and researchers, who should also involve adolescents in planning, implementing and assessing the services. There is a need for a self‐learning system to continuously adapt to user feedback.

**Conclusion:**

This study provides insight into adolescents' perspectives on the ideal mental health services. It offers suggestions for ways to improve services to better meet the individual mental health needs of adolescents. Additional research is needed to further develop and implement service changes, as well as to assess their acceptability, effectiveness, cost‐effectiveness and safety.

**Patient or Public Contribution:**

This is a study lead by adolescents. Adolescent coresearchers have, with the support of a senior researcher, planned and carried out the study, collected and analysed data and authored the manuscript.

## 1 INTRODUCTION

One in seven adolescents experiences a mental health disorder between the age of 10 and 19 years,^1^ increasing to one in five in the age group from 14 to 25 years.^2^ Adolescence is not defined by a standard age range. It is often understood as the period from 10 to 18 or 19 years, although in some instances up to the age of 25.^3^ Without early intervention and sufficient support, long‐term physical and mental health effects can persist into adulthood.^1^ However, up to 75% of adolescents with mental health problems do not seek help at an early stage or at all.^4^ Furthermore, 30%–75% of adolescents drop out of treatment.^5^ Previous studies found that the main barriers to service use include lack of knowledge about and stigma associated with mental health and the services, reluctance to speak to an unknown practitioner, concerns about confidentiality and practical obstacles such as long waiting lists and costs.^6^

Over the last decades, politicians, researchers and policy decision‐makers have developed the services with little or no input from adolescents. It was not until 2013 that the UK National Institute for Health and Care included public involvement as mandatory in healthcare development policies.^7^ The Public Health Association of Australia implemented the Community Participation Policy in 2019, requiring communication between healthcare authorities and users to ensure informed health service decisions.^8^ In recent years, youth have been consulted about healthcare services.^9^, ^10^, ^11^ The existing international literature primarily provides insight on user involvement at an individual level; less is known about involvement at the organizational level.^12^

### 1.1 Organization of mental health services

The organization of healthcare services varies from country to country. In some, services are free, but availability is reduced by long waiting lists. In other countries, services have shorter waiting lists, but are privatized and costly. Mental health policies, legislation and health programmes are used with the intention to improve the quality of mental healthcare systems. However, laws and guidelines for service provision, which comply with international recommendations and regulations, are mainly accessible in high‐income countries. Over 50% of people with severe mental disorders in these countries receive treatment, and governments spend an average of 2 USD per person annually on mental health services.^1^ Meanwhile, in middle‐ and low‐income countries, only 0.25 USD is spent per person each year, and 76%–85% of people with severe mental health conditions receive no treatment.

Examples of countries with differences in service provision include the United States, Australia, Mexico and Cuba. In the United States, some mental health services are provided for low‐income citizens; others must pay out of pocket. Privately insured adolescents account for nearly half of the expenditures and three‐quarters have unmet mental healthcare needs.^13^ In contrast, Australian low‐threshold healthcare services are provided nationally, free or at low cost.^14^ In Mexico, 45% of the population is covered by social security; others benefit from an insurance scheme.^15^ However, many hospitals and health centres lack personnel to cover treatment demand and mental healthcare institutions are lacking in rural areas. Mental health services in Cuba are free of charge and available to all.^16^ A UN Committee expert described the Cuban healthcare system to be one of the best globally.^17^

In Norway, mental healthcare is free and most adolescents who experience mental health challenges first contact a school nurse or a general practitioner. Low‐threshold outreach services are available for adolescents outside schools.^18^ Primary care services are the responsibility of municipalities.^19^ Since 2020, Norwegian municipalities are bound by law to offer psychologist services for mild to moderate mental health problems. Currently, 84% of municipalities provide such services.^20^ However, service provision varies considerably between the 356 municipalities due to variations in population size and budgets. Primary care practitioners can refer adolescents with more severe or persistent mental health conditions to child and adolescent psychiatric clinics for short‐ or long‐term care. However, the referral system creates a higher threshold to receive specialist help for mental health problems.

All children and adolescents in Norway have a legal right to access healthcare services of high quality.^21^ Pathways for mental health services were established in 2019 to secure adolescents' rights for timely and effective assessment and treatment.^22^ However, preliminary evaluations suggest that the pathways are inadequate and most practitioners do not find that it contributes to efficiently reaching treatment goals.^23^ Instead, it causes frustration and stress among healthcare practitioners, negatively influencing work motivation.

Existing evidence suggests that adolescents experience insufficient access to services. In our survey of 1500 adolescents, four in ten said that they would not talk to a healthcare practitioner about their mental health.^24^ Furthermore, 70% said that they had received insufficient information about the services available to them and more than half did not receive sufficient information about their own mental health issues.^25^ Moreover, GPs serve as gate‐keepers to the specialist mental health services. Only one‐third of adolescents who have been diagnosed with mental health conditions are referred for secondary care, even though GPs provide limited follow‐up.^26^ There is a significant gap between the services developed in theory by policymakers and what adolescents encounter in reality.

In 2019, the Norwegian Government established an escalation plan to support child and youth mental health.^27^ The plan stated that children and youth should, to a larger extent, be involved in service development. This is in line with the 2021 Patient and User Rights Act.^21^

The aim of this study was to explore adolescents' perspectives on the ideal healthcare services to meet their mental health needs.

### 1.2 The study background and context

This study was initiated and run by 10 adolescent coresearchers in the *InvolveMENT* research project, supported by the project lead (P. V.), who is a senior researcher. It is run by *SHARE—Centre for Resilience in Healthcare*, which was established in 2017 at the *Faculty of Health Sciences*, *University of Stavanger*, Norway. Patient and Stakeholder Involvement is one of *SHARE's* central strategic research priorities and the *InvolveMENT* project focuses on adolescent mental health, which has previously been described in *Health Expectations*.^28^ The coresearchers were invited to provide their perspectives on research priorities since project start. Within the first year, they also initiated, planned and carried out parts of the research, supported by the lead researcher.

The *InvolveMENT* project receives financial support for some activities through the *Resilience in Healthcare* project funded through the *Research Council Norway* (FRIPRO TOPPFORSK funding stream, grant agreement no. 275367).

### 1.3 Adolescents' contributions to the research

This study was initiated, planned and run by adolescent coresearchers. Adolescent seminar participants contributed with data, analysed and supplemented by the coresearchers. The manuscript has been authored by the coresearchers. The project lead has provided support throughout all phases and contributed as a coauthor.

## 2 METHODS

Qualitative methods were used to address the research question. Data were collected by and from adolescents. Analysis was carried out using a two‐stage approach: first, a qualitative content analysis, followed by a thematic analysis. This process was chosen to analyse the perspectives provided by 295 adolescents attending a mental health seminar (qualitative content analysis), followed by an exploration of 10 adolescent coresearchers' in‐depth understanding (thematic analysis).

The coresearchers and the lead researcher have worked closely together since 2017.^28^ This has included over 100 meetings with discussions focusing on youth mental health, service use, mental health research and research methods. This included some training workshops to support their research skills. Three coresearchers had previously planned and carried out cross‐sectional surveys in youth mental health, two were coauthors of a systematic review^12^ and all coresearchers were coauthors of a qualitative study, where their contributions to adolescent mental health research have been further described.^28^ Moreover, the coresearchers increased their academic writing and research skills as part of their academic education, which some started in 2018 and others in 2020. The lead author of the current study completed university psychology research methods courses, which also included training in qualitative content analysis. The researcher provided coresearchers with support throughout all phases of the research.

### 2.1 Participants and data collection

Data collection in Phase 1 took place at a 1‐day seminar on 26 September 2019 at the *University of Stavanger*. The seminar, entitled ‘We all have a mental health’, focused on the stigma associated with mental health and mental health services. It was organized by and for adolescents, and six out of seven seminar presentations were given by adolescents, focusing on mental health issues such as ‘suffering alone with depression’, ‘coming out of the closet’ and ‘having a refugee background’. Adolescents in lower (10th grade, age: 15–16 years) and upper secondary school (1st–3rd grade, age: 16–19 years) were invited to the seminar. An invitation was sent to all 72 schools in the county and the seminar was fully booked with 330 adolescents from 18 lower (*n* = 5) and upper (*n* = 12) secondary schools from all parts of the county, including cities and rural areas.

Participants could contribute verbally during presentations with comments and questions. They were also encouraged to provide anonymous written perspectives using an online tool—the Mentimeter app.^29^ Participants visited Mentimeter from their mobile devices and entered a seminar code to enable them to respond to the current questions. The tool supports active and equitable participation in discussions and data collection.^30^ It provided participants with an opportunity to share instant responses or reflect and respond by the time presentations ended.

Questions posed (Table 1) were used to explore adolescents' perspectives on the ideal healthcare services to meet their mental health needs. The questions had been prepared by the coresearchers with the support of the senior researcher. A total of 295 participants (89%) responded digitally to one or several questions. Response rates for individual questions varied from 17% to 55%. Responses were used for the qualitative content analysis.

**Table 1:** Questions posed during the seminar to explore adolescents' perspectives on the ideal healthcare services

1. What can you do if you feel isolated as a result of your mental health?^a^
2. What works well for adolescents struggling with their mental health in today's healthcare services?
3. What is missing to encourage more teens to seek professional help?
4. What can the healthcare services do for teenagers with a refugee background?
5. How should the school nurse^b^ be in order for you to seek their help when things are difficult?
6. What do you think is needed in order for it to be more ‘acceptable’ to seek help from a school nurse regarding mental health problems?

^a^ The first question was used to determine if adolescents would consider seeking help from the existing healthcare services.

^b^ Healthcare workers in schools.

Participants in the second stage were the 10 adolescent coresearchers.^28^ They were 16–20 years of age when they first joined the project and 17–22 years of age at the time of the seminar in 2019. They had then been involved in the project for one to two‐and‐a‐half years. They provided their perspectives based on preliminary categories identified through the qualitative content analysis. Summaries of coresearchers' descriptions were developed by three authors (L. G. M., L. K., P. V.) and further discussed and expanded by the full group. Meeting notes were made and used for further data analysis.

### 2.2 Data analysis

The qualitative content analysis was chosen to identify the trends and patterns of terms used by the seminar participants. The 295 participants contributed with 588 responses to questions used to explore their perspectives on the ideal healthcare services. Content analysis was used as an exploratory approach assessing large amounts of data, to map the frequency of data and patterns of responses (Table 2).^31^

**Table 2:** Data analysis Stage I—qualitative content analysis

Question	Categories
What can you do if you feel isolated as a result of your mental health?	Talk (*n* = 97). Participate in enjoyable activities (*n* = 31). Socialize (*n* = 20). Cry it out (*n* = 5).
What works well for adolescents struggling with their mental health in today's healthcare service?	Variety of services (*n* = 23). Understanding and acceptance (*n* = 14). Consistency (*n* = 13). Option for anonymous contact (*n* = 9). Social acceptance (*n* = 9). Easy access (*n* = 6). Taken seriously (*n* = 3).
What is missing to encourage more teens to seek professional help?	Improved availability (*n* = 34). Receiving relevant information (*n* = 30). Being understood and taken seriously (*n* = 9). Building trust (*n* = 6). Online support (*n* = 6).
What can the healthcare services do for teenagers with a refugee background?	Show kindness and acceptance (*n* = 29). Adapt communication (*n* = 20). Support integration (*n* = 11). Show interest and understanding (*n* = 9). Reach out to build a trusting relationship (*n* = 8). Provide a space for sharing experiences (*n* = 7). Expand cultural competence to work with refugee youth (*n* = 5). Provide support for anonymous contact (*n* = 2).
How should the school nurse be in order for you to seek their help when things are difficult?	Patiently listening and supporting (*n* = 25). Kind, accepting and caring (*n* = 23). Open and honest (*n* = 19).
What do you think is needed in order for it to be more ‘acceptable’ to seek help from a school nurse regarding mental health problems?	Information (*n* = 18). Reduce stigma (*n* = 13). Improve availability (*n* = 11). Listen and understand (*n* = 5).

In the second stage, the 10 coresearchers explored their own perspectives on the categories developed in Stage 1. A thematic analysis was used to enable in‐depth exploration of coresearchers' descriptions.^32^ Written notes of their reflections and discussions were in the form of narratives including context descriptions. Their reflections were assessed by three authors (L. G. M., L. K., P. V.), who wrote summaries for each category description. Categories were grouped into themes, each with a fuller description. The analytic process was iterative, as quotes from seminar participants and coresearchers were revisited and used to reassess the content of the themes until consensus on theme descriptions was reached. Notes were kept throughout the process.

Stage 2 of the data analysis provided more detailed and richer descriptions of the categories identified in the first stage. This provided better insight to understand how adolescents thought that help‐seeking could be improved and how drop‐out could be reduced. For example, a variety of services had been mentioned by several participants in the first stage. The data in the second stage analysis did, however, provide a richer description of the content of this category, which was therefore developed into a theme. As one of the participants stated: ‘A variety of services available for adolescents increases the chances for them to seek treatment. Not everyone is keen on […] talking face to face […] with a therapist, so having the ability to text and do it through call is important. […] Art therapy, animal therapy, music therapy, is also a good way of providing variety, and could allow for adolescents to help feel more comfortable during therapy if they do something enjoyable’. The data collected in the second stage also provided rich descriptions that brought to light how culture affected the other developed themes.

Participant quotes were used in‐text and in drawings to illustrate each theme. The drawings were developed by one of the coresearchers (M. I.) with input from the other coauthors.

## 3 RESULTS

**Figure 1:**
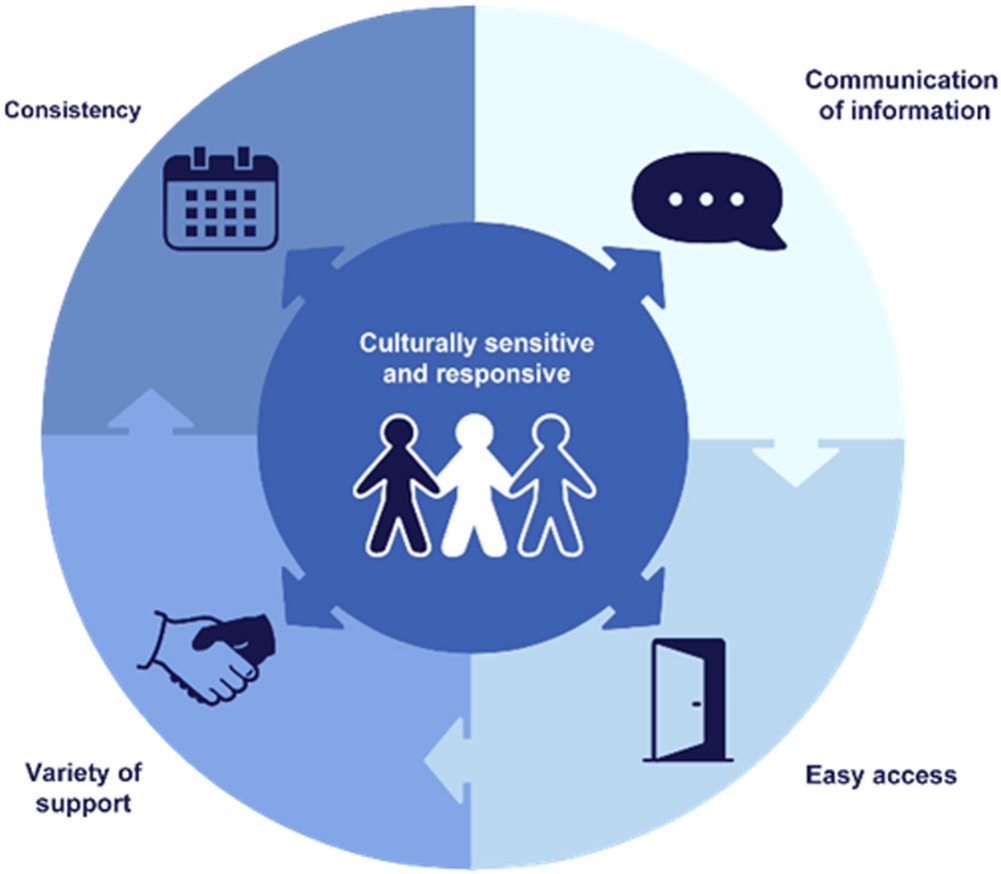
Model for the ideal adolescent mental health services

Five themes were developed to describe the characteristics of the ideal healthcare services for adolescents with mental health problems (Figure 1). The themes provide an insight into adolescents' perspectives on measures that can be taken to facilitate help‐seeking as well as to limit treatment drop‐out.

1. Culturally Sensitive and Responsive
2. Communication of Information
3. Easy Access
4. Variety of Support
5. Consistency

**Figure 2:**
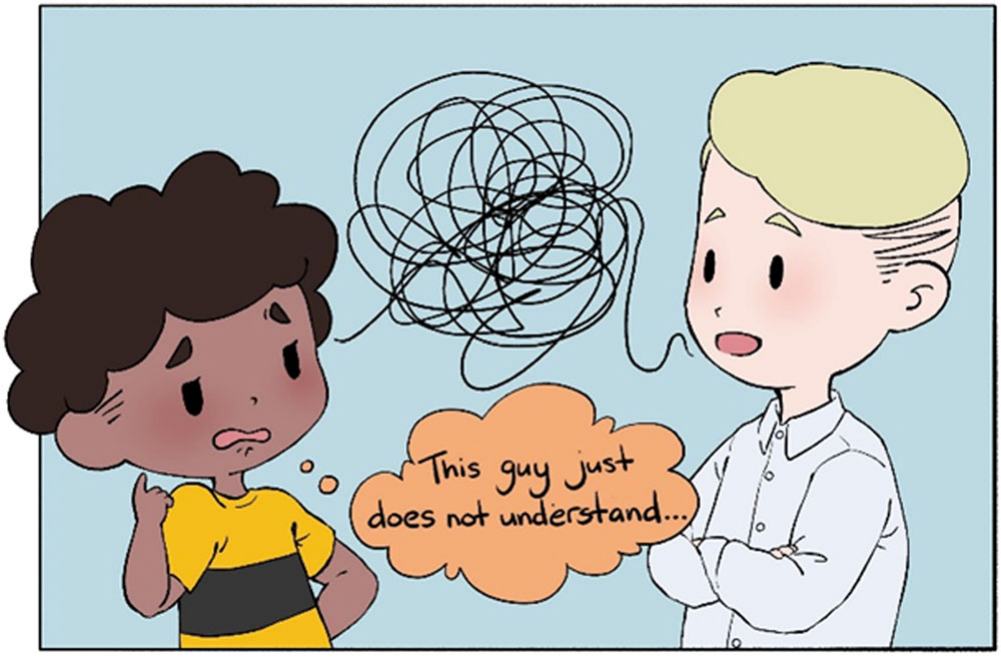
Culturally Sensitive and Responsive

The first theme, *Culturally Sensitive and Responsive services*, was considered essential for providing relevant healthcare services for adolescents with different experiences and backgrounds. It played an important role in each of the additional four themes (Figure 2).

### 3.1 Culturally Sensitive and Responsive

*I wouldn't have to explain so much if my therapist had cultural knowledge*. Figure 2

Adolescents' cultural background influence the way they relate to mental health and the healthcare services. For example, sharing private and personal information with a stranger may be considered more or less acceptable and associated with stigma/taboo in different cultures, instead of talking to family members or friends. It is important that the healthcare services are sensitive to adolescents' cultural backgrounds and respond appropriately to meet their individual mental healthcare needs.

To encourage adolescents to search for help, information needs to be easily available online in multiple languages, presented in both audible and illustrative ways aimed at different groups of adolescents, including those who are illiterate and the vision‐impaired. It is also important that the services actively reach out to adolescents who may be unfamiliar with mental health and unaware of the available options, for example, for newly arrived refugees.

Culturally competent translators should be available if adolescents and practitioners are unable to communicate through a shared language. Other forms of communication may sometimes be more helpful: ‘Healthcare practitioners [should be] ready to communicate with different methods, depending on what the adolescent is comfortable with, whether that is drawing, writing, or having a translator in the room’. Moreover, body language may have different meanings for adolescents from different cultures. Practitioners should be aware of their own body language and attempt to adapt to individual adolescents. If used constructively, body language can contribute to support verbal communication or feel reassuring where spoken communication is limited: ‘I remember my practitioner once held my hands and said: I am here for you, and will do anything for you to feel safe, but you need to trust me for me to help you. That's when I felt I was in safe hands’.

Where the adolescent and the practitioner are able to communicate, it is important to approach topics based on adolescents' knowledge of mental health. For example, adolescents from some cultures view mental health differently to a Western medical understanding and may, for example, consider such problems to be caused by spirits. Healthcare practitioners should be aware of this and be prepared to manage such conversations.

Different adolescents also have different experiences with and perceptions of mental health practitioners. For example, some believe that mental health practitioners are similar to general practitioners and expect a clinical medical treatment approach, whereas others seek guidance and prefer more collaboration. Their perceptions are in part influenced by their cultural background. Moreover, practitioners' physical characteristics and gender may also influence adolescents' willingness to use the services. For example, for some adolescents, it may be helpful to meet a practitioner of the same gender due to religious beliefs.

Overall, cultural identity is complex and formed by multiple factors such as ethnicity, race, religious and political views, education, peer and family influence, sexual and gender identity, to name a few. To varying degrees, each factor influences an adolescent's perspective on mental health and willingness to use the services. Although it is impossible to be aware of each adolescent's cultural background and needs before meeting them, being willing to adapt to their needs to enhance communication and build trust will aid in creating services that are experienced by adolescents as accessible and helpful.

### 3.2 Communication of Information

*Relevant information isn't just about where to get support, but also what do I do to receive help and are there options besides therapy?*

Services should provide relevant and easily understandable information, adapted to adolescents' cultural backgrounds. They should engage in dialogue with adolescents to adapt services to meet their mental health needs over time. ‘Many adolescents are willing to look for help but unable to due to the lack of information’. Adolescents need information about, for example:

1. What is mental health?
2. When should I reach out, and what if I need immediate help?
3. What happens if I reach out?
4. What is the first session like?
5. What services are available to me?

**Figure 3:**
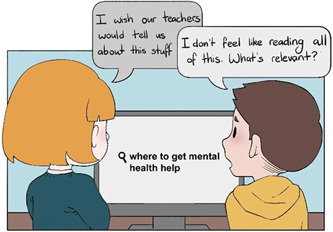
Communication of Information

Insufficient information may result in incorrect perceptions about the services and adolescents may feel uncertain and reluctant to seek help: ‘I was scared the services were in contact with the authorities and would share my private information’. Adolescents need access to easily understandable information from reliable online sources: ‘an overload of information that can make it difficult to sift out the relevant information, especially for someone whose energy is drained by mental problems’ (Figure 3).

Key mental health topics, including service information, should be taught in school and the complexity of the information should increase with age. Learning about mental health in school should be as natural as learning about physical health. This would also contribute to destigmatizing mental health. It is equally important for the services to reach adolescents who are not in school. This could be done through other means of communication such as social media, gaming networks and other out‐of‐school activities to reach adolescents where they spend a significant amount of their time.

Additionally, communication of information should be a two‐sided process—a dialogue. Adolescents need to experience healthcare professionals who listen attentively to them: ‘A lot of teenagers don't feel like they are taken seriously by adults. The more trusting the teenager is with the professional, the more they will feel comfortable with speaking to them. This will help them progress toward solving their mental health issues’. Dialogue is a necessary part to develop a trusting relationship that is essential to achieve progress in the long term and it can be used to continuously adapt the services to better meet adolescents' changing needs.

### 3.3 Easy Access

*The services have terrible opening hours… it should be open after school*.

**Figure 4:**
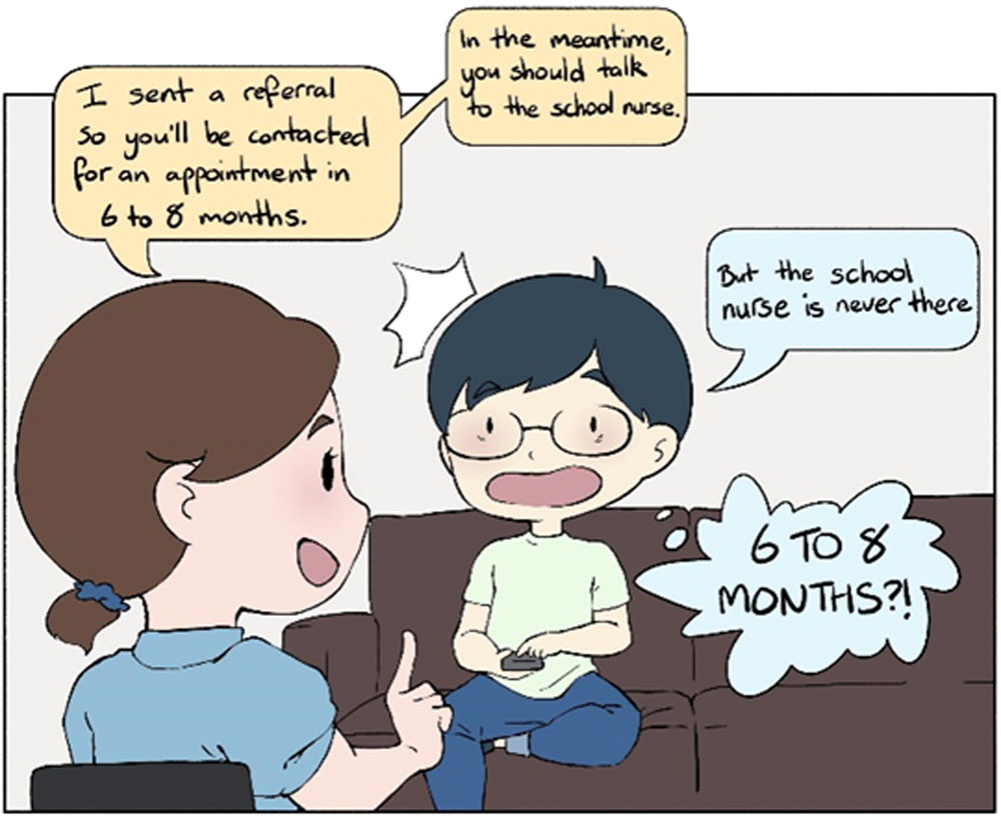
Easy Access, Part A

Accessible healthcare services are affordable, within reach, have short waiting lists and flexible hours. Many adolescents wait 6–12 months before their first appointment (Figure 4). By that time, their mental health can significantly deteriorate or they may lose hope and motivation to seek help. Quick access is possible with private practitioners, but costly, and not an option for those who have financial constraints or who do not want to involve their families.

Adolescents who attend school often experience that ‘the available hours of the school nurse is a running joke. Even when she is supposed to be there, she is often stretched thin over multiple schools and has to juggle other services’. Many do not know who their school nurse is and how they can help. Overall, the help‐seeking process can be discouraging. In order for adolescents to reach out, they ‘wish the nurse would come around and speak to them individually and in classes’, initiate conversations and provide clear and updated schedules.

**Figure 5:**
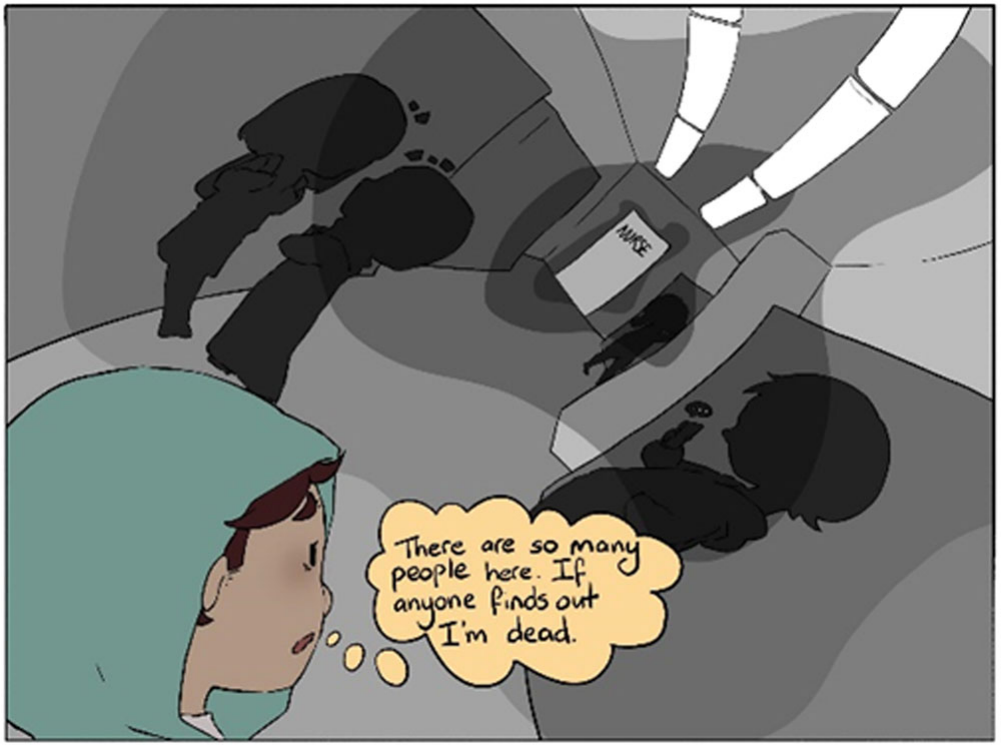
Easy Access, Part B

An online booking system should be available for all healthcare services. Access should also be timely in convenient locations. It should be placed so that they can be entered discreetly (Figure 5). Many prefer anonymous online services, where they can choose when to disclose information about their identity. Services must be accessible outside school hours and be user friendly. These aspects of service access are important to adolescents overall and may be even more important to those who have a minority background, ethnic or otherwise.

### 3.4 Variety of Support

*Tailor the support … don't put me in a box*.

Healthcare practitioners need to accommodate for individual needs early in the treatment process, rather than jumping to conclusions. By giving adolescents choices, practitioners communicate that they are actively listening and have genuine interest to help, creating a good start to build trusting relationships where both sides can communicate what they see as the most effective way forward.

**Figure 6:**
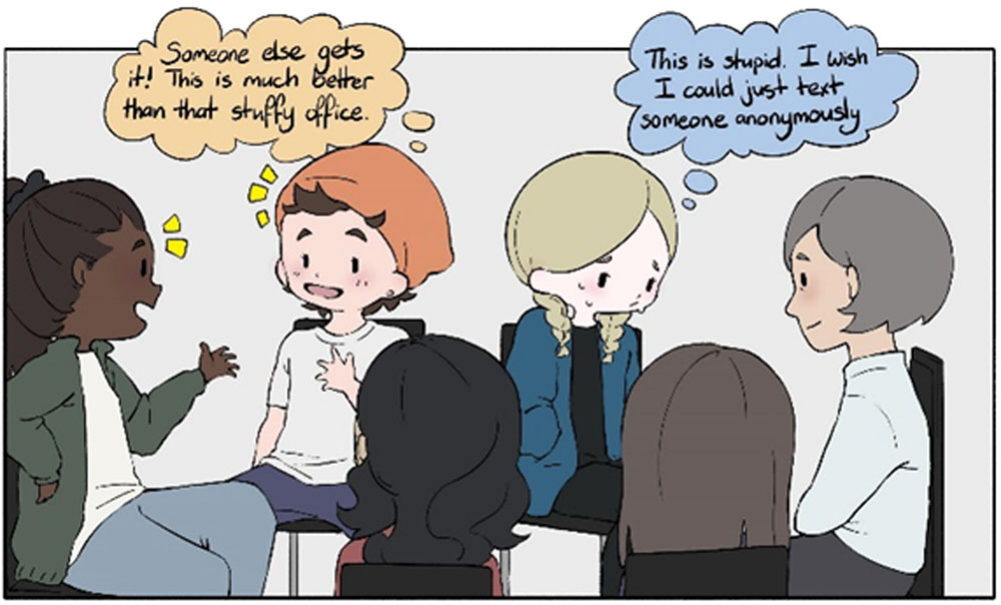
Variety of Support

In a technological society, texting practitioners or using online resources may correspond with how adolescents communicate in everyday life. Others prefer face‐to‐face meetings individually or group therapy with peers, which can also strengthen social bonds (Figure 6).

Moreover, how a practitioner meets an adolescent could be varied in an effort to ease the experience of seeking professional help, for example, asking if they prefer doing activities together, like simply making a cup of coffee in the office kitchen before sitting down. Offering a variety of activities can better meet individual needs and contributes to reducing tension and supporting openness. ‘Art therapy, animal therapy, music therapy, is also a good way of providing variety, and could allow for adolescents to help feel more comfortable during therapy if they do something enjoyable’. This may extend to the location of meetings. Sitting in an office is very formal, as opposed to going for a walk or sitting in an open area. Each individual will have preferences for support and treatment options that should be jointly considered by the practitioner and the adolescent, where the adolescent's cultural background should also be taken into consideration.

### 3.5 Consistency

*It took months before I wanted to share my experiences with my therapist*.

Service consistency means that adolescents should experience little unanticipated changes in the care that they receive. ‘Adolescence is a tumultuous period of life with many changes… healthcare services that are consistent are extremely important [to] ensure they get the help they need’.

**Figure 7:**
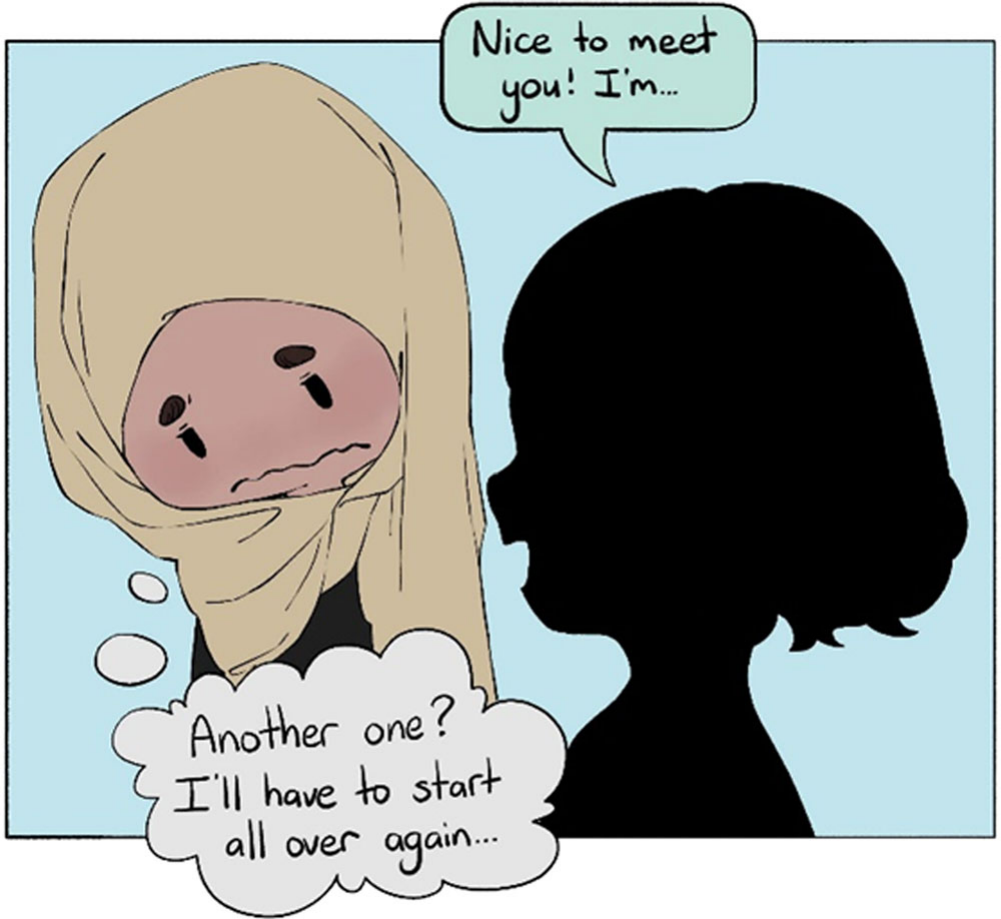
Consistency

Raised concerns include ‘the therapists changing too often, making it difficult to progress’. Adolescents are then forced to explain the same issues to different practitioners, over and over again. This is discouraging and they may withhold information to avoid repeating themselves (Figure 7). It is demotivating and halts progress towards achieving mental well‐being, along with the adolescents repeatedly being reminded of the trauma without consistent support from one practitioner. This may be even more detrimental for adolescents with minority backgrounds, some of whom may have significant difficulties in trusting ‘strangers’.

Over time, adolescents may limit their openness and willingness to try practitioners' suggestions and therapy. They do not feel that practitioners know ‘the whole story’ and they are therefore unequipped to help. On the other hand, adolescents may also wish to change the practitioner. Although practitioner change is permitted, it is not consistently offered. While some adolescents can request changes, others are reluctant to do so or are met with ‘doubt and opposition’. This causes some to drop out of treatment.

Adolescents need a system that reinforces trust, and encourages hope and progression. Adolescents who feel comfortable with the practitioner are more likely to approach therapy with a positive mindset and build long‐term, trusting relationships. They are more open to feedback, support and suggestions. Regular appointments agreed ahead of time can facilitate scheduling their busy lifestyles around appointments, thereby reducing the likelihood of dropping out.

## 4 DISCUSSION

This study explored adolescents' perspectives on the ideal healthcare services to meet their mental health needs. *Culturally Sensitive and Responsive* services played an important role in the other four themes: *Communication of Information*, *Easy Access*, *Variety of Support* and *Consistency*.

In an increasingly globalized and multicultural society, practitioners must recognize the complexity of individual adolescents' cultural identity, and provide support accordingly, to better meet their expectations and needs.^33^ Culturally sensitive services have ‘the ability to be appropriately responsive to the attitudes, feelings, or circumstances of groups of people that share a common and distinctive racial, national, religious, linguistic, or cultural heritage (p. 131)’.^34^ Eight years ago, The Lancet Commission expressed a need for a more descriptive definition of culture and ‘making culture central in care practices (p. 1608).’^35^ We challenge current views of cultural identity and suggest that an updated and extended understanding should be considered. Culturally sensitive mental health services should no longer be limited to adolescents' ethnic backgrounds, nationality, race and religion. Today, cultural identity can be influenced by subcultures such as fitness,^36^ LGBTQ+^37^ and online gaming cultures.^38^ Along with rising immigration rates and the exponential growth in social media use, adolescents are consistently introduced to new cultural experiences that shape their views, likes and dislikes. How do these different influences affect their understanding and perception of the world and how does it affect their mental health? For example, how would an adolescent be influenced by originating from Russia, living in Switzerland, preferring Western music and Asian beauty standards, being engaged in U.S. politics and guided by astrological readings? Such line of questioning is in line with others who point out that ‘people are cultural beings whose histories, values and experiences shape their understanding of what constitutes good or normative behaviour and how they make sense of the world (p. 11406)’.^39^

Healthcare practitioners cannot possibly possess knowledge about and understanding of all cultures and subcultures. However, they can develop skills through training and experience to *sense* individual differences in adolescents, approach them with *interest* and *respond* by adjusting treatment and support to better meet their needs. Although national policies such as the US Office of Minority Health's national guidelines for minority health have existed for two decades, our study suggests that there is a need for greater focus on the *cultural responsiveness* of the services. Support and treatment should be better adapted to meet individual needs.

The Norwegian government issued a youth mental health escalation plan in 2019. It presents a 5‐year plan to adjust services to meet adolescents' needs. Since then, specialist healthcare services, schools and social clubs have been adapted to better align with the culture of indigenous (Samí) youth.^40^ For youth with immigrant backgrounds, the Government's plan is mainly limited to reducing language barriers. This includes training and use of translators and recruiting persons with ethnic minority backgrounds to become healthcare practitioners. However, it is unrealistic to aim for specialized practitioners or translators in each of Norway's 356 municipalities, with anywhere from 200 citizens to 700,000 inhabitants, to address all adolescents' needs. A much better solution would be to train all healthcare practitioners to enable them to provide culturally sensitive and responsive services irrespective of adolescents' background. Additionally, some therapists with more extensive cultural competence could serve as supervisors to provide guidance for other healthcare practitioners. The latter has, to some extent, been implemented by the *Transcultural Centre* at *Stavanger University Hospital* ^41^ and the *Regional Centre for Violence, Traumatic Stress and Suicide Prevention*, with increased engagement of youth.^42^ Unfortunately, their resources are limited and their services are insufficient to support healthcare practitioners nationwide.

Our research suggests a need for a system of services that ensures *Communication of Information*, *Easy Access*, *Variety of Support* and *Consistency*. Each of these themes is also affected by *Culturally Sensitive and Responsive* services. In particular, professionals' cultural competence is essential to enable *Communication of Information* to facilitate mutual understanding between healthcare professionals and adolescents. However, *Culturally Sensitive and Responsive* services are also important for the remaining three themes. Communication alone is insufficient if the services are not perceived as accessible by adolescents. *Easy Access* can, for example, require flexible opening hours and convenient location of services to enable adolescents to discreetly enter clinics, which may be particularly important for those coming from cultures where use of mental health services is strongly associated with stigma. Similarly, *Variety of Support* is important to adapt to individual adolescents' mental healthcare needs, which can be strongly influenced by their cultural background. For example, group therapy may be unthinkable within the context of some cultures. Finally, adolescents' cultural background may also influence their expectations of the *Consistency* of services. Change of therapists or limiting treatment to 10 sessions, as provided within the context of the current mental health pathways in Norway, might increase reluctance to seek help or contribute to early drop‐out.

Others also found that adolescents report being unaware of where they can receive information or help.^6^, ^43^ Along with concerns that practitioners might breach confidentiality^44^ and negative expectations about the treatment,^6^, ^45^ adolescents may be unaware of potential benefits of available treatment and support. The *Communication of Information* theme showed that to avoid misinformation, there is a need for open and inclusive dialogue at a microlevel, between individual adolescents and their practitioners; at a mesolevel, between groups of adolescents and the services; and at a macrolevel with decision‐makers. However, communication of information is not limited to teaching adolescents about mental health in schools. It is a two‐sided conversation where adolescents are also *consulted* about the services, demonstrating that service providers are actively attempting to provide relevant support, in turn contributing towards building adolescents' trust in the healthcare system. As a result, adolescents might feel more motivated to reach out to the services and to meet healthcare practitioners with positive expectations. Adolescents are also more likely to utilize the services when they take the initiative to seek help.^6^, ^46^ By providing sufficient and relevant information about mental health, whether through direct communication with adolescents or using posters with general information, including about when and where they can seek help, adolescents will be better equipped to reach out when needed. It is also important that the main focus of mental health teaching in school is mental wellness, for example, to learn simple self‐help approaches to manage everyday challenges.

Moreover, motivation to *access* the services is closely linked to stigma.^6^ Over time, stigma could be reduced through mental health awareness provided through schools and in other areas of everyday life, such as soccer coaches including mental well‐being when talking about healthy habits. Current services should adapt by meeting adolescents where they feel comfortable and reducing stress factors linked to contacting the services.^41^ For example, having texting options to limit concerns that schoolmates might question adolescents' absence from class; meeting them outside for a walk to reduce the feeling of a ‘doctor's visit’; or offering youth to bring a support person of their preference could contribute to feeling more secure with their practitioner. Our suggestions for actions to address service use barriers are in line with those previously identified.^6^

Adolescents are more likely to seek help when they feel respected, listened to and not judged.^6^ However, individual adolescents' needs vary. For example, they need a *Variety of Support* outside the standard one‐on‐one sessions with a therapist. Many feel uneasy when sharing personal information with a stranger in a ‘conventional’ therapy setting.^6^ The best approach to build trust varies. Some adolescents prefer a clinical one‐on‐one approach; others prefer group activities. A shared journal for adolescents to use in between their appointments, where both the adolescent and the practitioner have access, could be beneficial for those who struggle to remember things that they want to share. It would also give practitioners first‐hand insight into the adolescents' experiences, and adolescents who struggle to find a starting point during their session could reflect back and decide what was most important to them. Varied support could include a broad range of approaches, for example, creating online computer games that allow for communication with healthcare practitioners or other adolescents.^47^ Other options could be to provide adolescents with brief descriptions of practitioners with their hobbies and interests, to make meeting them less daunting, or set up voluntary after‐school group activities, such as simply making a camp fire with a guidance counsellor discussing mental wellness and encouraging adolescents to speak openly about their everyday problems with peers and a practitioner when they need it.

Finally, adolescents also need *Consistency* of the services to strengthen the therapeutic relationship, to provide support networks and a ‘way forward’ into adulthood. Previous studies support our findings suggesting that adolescents prefer having a single practitioner to relate to. They can experience repeated changes of practitioners as demotivating.^48^, ^49^ Studies reporting on continuity of care focus mainly on the transition from child and adolescent services to adult services. A limited number of studies explore the importance of continuity outside these transitional stages. Others found that continuity is often limited to the relationship between the adolescent and their practitioner; meanwhile, youths' reported experience of continuity was dependent on multiple life factors.^50^ Youth have a better experience of continuity when they are regularly informed, heard and included in the development of their treatment plans. Our findings suggest that consistently meeting the same practitioner contributes towards building trust and a chance to open up. However, practitioner consistency is only relevant when the adolescent experiences a positive quality of care with the practitioner over time. This includes factors like feeling heard and being involved and extends to having consistency in the scheduled meetings to be able to plan school, social and extracurricular activities.

On the other hand, too rigid routines may become a limiting factor for adolescents' motivation to continue treatment if the relationship is perceived as unproductive. Rigid routines and lengthy processes with limited options for changing practitioners can lead to adolescents dropping out from treatment all together. To ensure a positive outcome from consistent services, it is important that a good relationship is built between the practitioner and youth first so that the youth can trust their practitioner.

The themes identified through this study are equally important in providing a system that is available to each adolescent, regardless of personal needs and background. But how are they interconnected? For example, adolescents who have recently moved to Norway: What information will they need about the available mental healthcare services and how will they obtain this information? What can be done to ensure that they have easy access to the services? Do the services need to reach out to them? What type of services are needed to meet their needs? Are they confident that the therapist will be there for them over time? Are they able to change the therapist if they are not compatible? Are any parts of these aspects in conflict with their cultural identity? If any of these aspects are not up to their expectations and needs, they are more likely not to seek help or engage in a longer‐term therapeutic relationship. If, on the other hand, the services are in line with their mental health service needs, then they are more likely to experience the benefits of engaging with the services.

### 4.1 Implications of the research

We suggest that implementation of the proposed measures described as part of the five themes may increase the likelihood of adolescents seeking help from the mental health services and reduce treatment drop‐out.

The research has multiple implications for practitioners, educators, system organizers, researchers and adolescents. We suggest that the current healthcare system needs to be redesigned through the involvement of multiple stakeholders, including researchers, service designers, technology experts, clinicians, mental health/interest organizations and last but not least adolescents and their support networks (e.g., family members and friends). We suggest adolescents should take part as co‐researchers in planning and carrying out the research and as participants in testing the implemented changes. Suitable research designs could include e.g. approaches to codesign/coproduction and action research, in particular, with a view to also integrating culturally diverse perspectives.^51^, ^52^

Educational institutions should teach healthcare students to be sensitive and flexible to adolescents' individual differences and how to adjust the service to meet their needs. Teachers and schools should encourage adolescents to play an active role in their mental health by teaching them about mental wellness. Healthcare practitioners should contribute to establish a trusting relationship, to encourage hope and engagement. Institutional leaders should consistently check with service users to gather their perspectives. Politicians and policymakers should acknowledge the importance of youth mental health and allocate sufficient funds.

Moreover, implemented changes to the healthcare services should be assessed using rigorous research methods to determine the acceptability, effectiveness, cost‐effectiveness and safety of the services, using pragmatic (cluster‐)randomized‐controlled trials, case–control and cohort studies. This should be combined with qualitative research methods, in particular, to gain insight into adolescents' and healthcare practitioners' experiences with the implemented changes. We suggest that a revised and improved mental health service should also be a self‐learning system, that is, it should include strategies to further learn from the on‐going experiences gained within the services.

Redesigning the mental health services and implementing a continuously learning and changing service system could contribute towards significantly improving mental health in adults as well. One in eight adults is affected by mental disorders.^53^ Three in four experience mental health symptoms before the age of 24.^54^ Improved awareness and access to properly equipped services for youth will enable prevention, early detection of mental health problems and effective provision of interventions.

### 4.2 Strengths and limitations

The main strength of this study is that the research was planned, led and carried out by adolescent coresearchers. It is not unreasonable to assume that the anonymized and adolescent‐to‐adolescent data collection approach may have contributed to honest responses. Adolescent co‐researchers leading the project likely strengthens the relevance of the results to other adolescents. On the other hand, their limited research competence could pose a potential risk to the validity and reliability of the results. However, a senior researcher was extensively involved in planning the project, analysing the data and coauthoring the manuscript. The involvement of healthcare practitioners would have been likely to provide different perspectives, but the focus of this study was to emphasize the voices of adolescents.

The data in this project were collected from adolescents in lower and upper secondary school, and therefore, results may not be generalizable to the population of younger adolescents or young adults, as well as those who have dropped out of school. Moreover, caution should be exercised when generalizing results to other countries due to cultural differences and different systems of health service provision.

## 5 CONCLUSION

This study offers an insight into the characteristics of the ideal healthcare services to meet adolescents' mental health needs, viewed from their own perspectives. The results highlight the importance of providing services that are culturally sensitive and responsive. To adapt the healthcare services within the context of a pluralist culture requires consideration of multiple aspects of culture. It challenges an understanding of culture limited to race and ethnicity. Such adaptation of services has direct consequences for communication of information as well as easy access, variety and consistency of services. Further research is needed to determine how to implement the suggested proposals as part of revising and renewing the existing healthcare services. Using the findings in this study as part of service improvement can potentially contribute towards increasing treatment uptake and reducing drop‐out among adolescents who face mental health challenges. It can therefore contribute towards strengthening the quality of services and safety for adolescents.

## AUTHOR CONTRIBUTIONS

All authors were involved in planning the study. Phase 1 data collection was carried out by Maya Ibenfeldt during a mental health seminar organized and run by Maren McLean Andvik, Laia G. Meldahl, Lou Krijger, Oliver Cuddeford, Samuel Duerto, Maya Ibenfeldt, Murad Mustafa, Mathias Tong and Petter Viksveen. Phase 1 data analysis was carried out by Laia G. Meldahl, with support from Petter Viksveen. All authors were involved in Phase 2 data collection and data analysis in the first stage of Phase 2. Laia G. Meldahl and Lou Krijger carried out data analysis in the second stage of Phase 2 data analysis, supported by Petter Viksveen. Laia G. Meldahl and Petter Viksveen wrote the first draft manuscript, with input from Lou Krijger. Maya Ibenfeldt developed all figures, with input from the other authors. All authors contributed to the revision of the manuscript and approved the final version.

## CONFLICT OF INTEREST

The authors declare that there is no conflict of interest.

## ETHICS STATEMENT

The data collected in Stage 1 of this study project were anonymized (no direct or indirect person identifiable data), whereas data in Stage 2 included the adolescent coresearchers' own perspectives. No ethics approval was therefore required.

## Data Availability

The data of the study are available upon reasonable request (partly in Norwegian, partly in English).
